# Lowered Expression of Tumor Suppressor Candidate *MYO1C* Stimulates Cell Proliferation, Suppresses Cell Adhesion and Activates AKT

**DOI:** 10.1371/journal.pone.0164063

**Published:** 2016-10-07

**Authors:** Kittichate Visuttijai, Jennifer Pettersson, Yashar Mehrbani Azar, Iman van den Bout, Charlotte Örndal, Janusz Marcickiewicz, Staffan Nilsson, Michael Hörnquist, Björn Olsson, Katarina Ejeskär, Afrouz Behboudi

**Affiliations:** 1 School of Bioscience, Tumor Biology research group, University of Skövde, SE-541 28, Skövde, Sweden; 2 Department of Medical and Clinical Genetics, Sahlgrenska Academy, University of Gothenburg, SE-405 30, Gothenburg, Sweden; 3 Department of physiology, Faculty of Health Sciences, University of Pretoria, Pretoria, 0007, South Africa; 4 Department of Pathology, Sahlgrenska University Hospital, SE-413 45, Gothenburg, Sweden; 5 Department of Obstetrics and Gynecology, Halland Hospital Varberg, SE- 432 37, Varberg, Sweden; 6 Institute of Mathematical Statistics, Chalmers University of Technology, SE-412 96, Gothenburg, Sweden; 7 Department of Science and Technology, University of Linköping, ITN, SE-601 74, Norrköping, Sweden; CHA University, REPUBLIC OF KOREA

## Abstract

Myosin-1C (*MYO1C*) is a tumor suppressor candidate located in a region of recurrent losses distal to *TP53*. Myo1c can tightly and specifically bind to PIP2, the substrate of Phosphoinositide 3-kinase (PI3K), and to Rictor, suggesting a role for MYO1C in the PI3K pathway. This study was designed to examine MYO1C expression status in a panel of well-stratified endometrial carcinomas as well as to assess the biological significance of *MYO1C* as a tumor suppressor *in vitro*. We found a significant correlation between the tumor stage and lowered expression of MYO1C in endometrial carcinoma samples. In cell transfection experiments, we found a negative correlation between *MYO1C* expression and cell proliferation, and *MYO1C* silencing resulted in diminished cell migration and adhesion. Cells expressing excess of MYO1C had low basal level of phosphorylated protein kinase B (PKB, a.k.a. AKT) and cells with knocked down *MYO1C* expression showed a quicker phosphorylated AKT (pAKT) response in reaction to serum stimulation. Taken together the present study gives further evidence for tumor suppressor activity of *MYO1C* and suggests MYO1C mediates its tumor suppressor function through inhibition of PI3K pathway and its involvement in loss of contact inhibition.

## Introduction

A minimal region of recurrent deletion/allelic loss distal to the *Tp53* gene was identified in an experimental rat model for endometrial carcinoma [1]. Deletions at the homologous position on human chromosome 17 (17p13.3) unassociated with *TP53* mutation have been reported in several types of human tumors [2–5], suggesting a tumor suppressor activity in this chromosomal region. In deletion mapping, combined with gene expression, sequencing and epigenetic silencing, the candidate region was delimited and *Myo1c* was identified as one of the most likely candidates for the proposed tumor suppressor activity [6].

*MYO1C* encodes a class I unconventional myosin [7, 8], which is implicated, among other possible functions, in angiogenic signaling [9], glucose uptake [10–12] and the progression of the cell cycle [13]. The *MYO1C* gene is located on human chromosome 17 and encodes three isoforms of myosin-1c protein, two of which are found in the nucleus and cytoplasm, while the third is found exclusively in the nucleus [14–16]. Myo1c can bind tightly and specifically to PIP_2_ (phosphatidylinositol 4,5-bisphosphate) and InsP_3_ (inositol 1,4,5-trisphosphate) seemingly through its putative pleckstrin homology domain [17, 18]. When PIP_2_ localizes to lipid rafts in podocytes, myosin-1c becomes spatially associated with lipid rafts through this tight binding [19]. PIP_2_ is an important second messenger involved in some crucial cellular functions, including the regulation of the cytoskeleton and vesicle movements [20]. Myo1c is essential for the trafficking, translocation and fusion of exocytic GLUT4 (glucose transporter type 4)-containing vesicles with the plasma membrane upon insulin stimulation in muscle and adipose tissue [10–12, 21]. Depletion of Myo1c or over-expression of dominant-negative forms of the protein impaired this function in mouse fibroblasts [10, 22]. Insulin-stimulated phosphorylation of Myo1c is important for translocation of GLUT4-vesicles [23, 24] as well as for docking or fusion of GLUT4-vesicles to the membrane through reinforcement of Myo1c binding with the PI3K/AKT signaling pathway regulatory 14-3-3 protein that is associated with an increased ATPase activity of Myo1c [23, 25]. There is no earlier report on potential tumor suppressor activity of *MYO1C*; however, another member of the myosin-I gene family, *MYO18B*, has been recognized as a tumor suppressor gene candidate in lung, ovarian and colorectal cancer [26, 27].

Deregulation of the PI3K/AKT pathway is a recurrent feature in numerous human malignancies with a key role in cancer development, progression and also in resistance to chemotherapy [28]. Over-activity of this pathway is commonly caused by loss of function of the tumor suppressor gene *PTEN* [29, 30], oncogenic activation of *PIK3CA* [31, 32] and/or over-stimulation by various growth factors, namely IGF-1, EGF or VEGF [33–35]. Although roles of MYO1C in the insulin-mediated signaling for glucose receptor transport are well established, details of its potential involvement in cancer development through the PI3K/AKT signaling pathway remain to be investigated.

In the present work, we examined the level of MYO1C in a panel of well-stratified endometrial carcinomas to inspect the potential correlation of MYO1C protein levels with tumor stage and prognosis. Our analysis showed a significant negative association between MYO1C protein level and the endometrial carcinoma tumor stage. To investigate the potential mechanism/s involved, we performed cell transfections for MYO1C protein over-expression and/or knockdown followed by cell proliferation, cell migration, and cell spreading/adhesion assays to investigate the potential contribution of *MYO1C* to these cancer phenotypes. As earlier works suggest for potential involvement of MYO1C in the PI3K/AKT pathway, we additionally performed protein level analyses for a number of key components of the PI3K/AKT signaling pathway in cells with over-expression or lowered expression of *MYO1C* in order to investigate the nature and consequence of involvement of MYO1C in PI3K/AKT and RAS/ERK signaling. Our analysis revealed a negative correlation between levels of MYO1C protein level and activation of PI3K/AKT signaling and cell proliferation. Our analysis additionally showed that lowered expression of *MYO1C* resulted in impaired cell migration and cell adhesion.

## Material and Methods

### Immunohistochemistry analysis of human endometrial carcinomas

All experiments on human tumor samples were approved by the local ethical committee (Sahlgrenska Academy, University of Gothenburg). All participants provided their written informed consent to participate in the study, all documents are archived at Sahlgrenska University Hospital; This procedure was approved by the ethical committee. A total of 62 endometrial carcinomas– 19 highly differentiated stage I tumors, 24 moderately differentiated stage II tumors, 19 poorly differentiated stage III tumors, and 10 endometrial hyperplasias–were analyzed. The tumor samples were randomly selected based on their pathology from material in the tissue bank of paraffin blocks. Immunohistochemistry was performed on paraffin sections from the samples above, using conventional staining methods in an EnvisionR system. The slides were pretreated with Tris-EDTA and stained with 1:50 rabbit anti-SKIP (S8948, Sigma Aldrich), 1:3000 rabbit anti-MYO1C (HPA001768, Sigma Aldrich). A section from lymphoid tissue was used as a positive control for MYO1C. The slides were evaluated using conventional light microscopy, regarding the following parameters: localization in cells of positive staining, level of positivity (0-1-2-3-4) in the tumor cell population, and fraction of positive cells in the tumor cell population. Corresponding slides stained with haematoxylin-eosin were available to determine areas of tumor in the sections.

### Cell lines and cell culture

**Fig 1:**
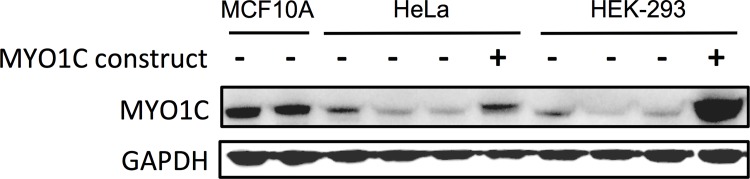
Expression of *MYO1C* in human embryonic kidney HEK-293, cervical cancer HeLa, and normal breast epithelium MCF10A cell lines. Immunoblot of MYO1C protein was performed in embryonic non-cancerous HEK-293, adult non-cancerous MCF10A and adult cancerous (HeLa) cell lines. HeLa and HEK-293 cells transfected with MYO1C expression construct were included as positive control and also to examine the specificity of the MYO1C antibody. *De no*vo expression of MYO1C was found to be low in the embryonic HEK-293 and the cancerous HeLa cells, and high in the normal breast epithelial MCF10A cells. Three independent experiments were performed and the figure shows a representative of these repeats.

HEK-293 cells (human embryonic kidney cell line, American Type Culture Collection, ATCC, USA) and HeLa cells (Henrietta Lacks’ cervical cancer cell line, Sigma-Aldrich/The European Collection of Cell Cultures, ECACC, UK) with limited *de novo* expression of MYO1C (Fig 1) were used for gene expression transfection studies. The cell lines were cultured in DMEM medium (21063–029, GIBCO by Life Technologies, USA), supplemented with 10% bovine growth serum (BGS; SH30541.02, Hyclone by Thermo Scientific, USA), and 1% penicillin and streptomycin (PEST; 100X; P11-010, PAA Laboratories, Pasching, Austria).

For the siRNA gene silencing experiment, the immortalized normal breast epithelium MCF10A cells (Michigan Cancer Foundation, ATCC, USA) with medium to high *de novo* expression of MYO1C (Fig 1) were cultured, as described earlier [36], in DMEM/F12 medium (31330–038, GIBCO by Life Technologies), supplemented with 5% horse serum (16050–122, GIBCO by Life Technologies), 20 ng/ml epidermal growth factor (EGF; AF100-15, Peprotech, USA), 0.5 mg/ml hydrocortisone (H-0888, Sigma-Aldrich), 100 ng/ml cholera toxin (C-8052, Sigma-Aldrich), 10 μg/ml insulin (I-1882, Sigma-Aldrich), and 1% penicillin and streptomycin (PenStrep; 100X; 15070–063, GIBCO by Life Technologies). All cells were cultured in the atmosphere of 5% CO_2_ at 37°C.

### Plasmid DNA and cell transient transfection

Gene expression construct for *MYO1C* (TrueORF cDNA clones and PrecisionShuttle vector system, SC 315975) and a corresponding empty vector, pCMV6-XL5 (PS100020) were purchased from OriGene Technologies, USA. Bacterial transformation was performed using *Escherichia coli* as host (*E*. *Coli*, NovaBlue Singles Competent Cells, Novagen by Merck KGaA, Germany). Midiprep Kit (Qiagen, USA) was used for plasmid purification.

Transient transfection was performed using Lipofectamine 2000 (11668019, Invitrogen by Life Technologies) prepared in Opti-MEM I reduced serum medium (11058, GIBCO by Life Technologies). Cells were transfected with four different mixtures of *MYO1C* gene expression construct and the corresponding empty vector so that all cells always received a maximum of 0.9 μg/ml external DNA. The four different gene construct concentrations were prepared as follows: 0.9 μg/ml empty vector + 0.0 μg/ml *MYO1C*-construct, 0.6 μg/ml empty vector + 0.3 μg/ml *MYO1C*-construct, 0.3 μg/ml empty vector + 0.6 μg/ml *MYO1C*-construct, and 0.0 μg/ml empty vector + 0.9 μg/ml *MYO1C*-construct.

For cell proliferation assays, the final ratio of total plasmid DNA to Lipofectamine 2000 was 0.09 μg to 0.45 μl per well of 96-well plate (167008, Nunc by Thermo Scientific); for protein expression analyses, 3 μg to 10 μl per well of 6-well plate (140675, Nunc by Thermo Scientific) and 6 μg to 15 μl in 60-mm petri dish format (150288, Nunc by Thermo Scientific).

### siRNA transfections for gene knockdown studies

siRNA duplexes (MYO1CHSS106886) for *MYO1C* knockdown and corresponding scrambled siRNA duplexes (medium GC contents, 12935–300) as the negative control were purchased from Invitrogen by Life Technologies. siRNA duplexes for *MYO1C* were sense: 5’-CCUAUCGCCGCAAAUACGAAGCUUU-3’, antisense: 5’-AAAGCUUCGU AUUU-GCGGCGAUAGG-3’. siRNA transfections for *MYO1C* knockdown were performed using Lipofectamine RNAiMAX (1378–075, Invitrogen by Life Technologies) in Opti-MEM I reduced serum medium as described by the manufacturer.

For cell migration assay, SMARTpool siGENOME siRNA for MYO1C (M-015121-0005) and siGENOME Non-Targeting siRNA Pools (D-001206-14-05) were purchased from Dharmacon by Thermo Scientific, USA. The pool of siRNA for *MYO1C* contains 1) sense: 5’-GGCUACAAGCCAGAAGAGUAC-3’, 2) sense: 5’-UCAGUUACCTCCT GGAAAAG-U-3’, and 3) sense: 5’-GCUCAAAGAAUCCCAUUAUGA-3’. siRNA-transfection was performed using DharmaFECT1 (Thermo Scientific, USA) as described by the manufacturer.

### Cell proliferation assay

The effects of over-expression and knockdown of *MYO1C* on proliferation was performed in HEK-293 and MCF10A cells, respectively. The final concentration of *MYO1C*-construct used in the over-expression experiment was mentioned earlier; the final concentration for *MYO1C*-siRNA in the knockdown experiment for cell proliferation assay was 10nM per well. Results from these assays were analyzed using 3-(4,5-dimethyl-2-yl)-5-(3-carboxymethoxyphenyl)-2-(4-sulfophenyl)-2H-tetrazolium compound or the so-called colorimetric MTS-based assay. In brief, cells were seeded into 96-well plate at 1x10^4^ cells per well for HEK-293 or 0.75x10^4^ cells per well for MCF10A and were subjected to transfections. At different time points post transfection (24, 48, 72 or 96 hours), 20 μl of the CellTiter 96 AQueous One Solution (G3582, Promega, USA) was added into each well containing 100 μl of growth medium, cells were incubated for 2.5 hours and the quantity of metabolized formazan, which is directly proportional to the number of living cells in culture, was measured by 490 nm-absorbance and recorded using Mithras LB940 (Berthold Technologies, Bad Wildbad, Germany) with the program MikroWin v.4.31 (Mikrotek Laborsystems, Overath, Germany). At least three independent experiments, each in quadruplicates, were performed.

### Cell migration assays

MCF10A cells were seeded in 6-well plates and transfected after 24 hours with either scrambled siRNA, serving as a control, or Dharmacon siRNA for *MYO1C* (M-015121-005, Thermo Scientific) to a final concentration of 20 μM (as recommended by the manufacturer) using DharmaFECT1 (Thermo Scientific). After incubation for 24 hours, cells were trypsinized, counted, diluted to 2x10^5^ cells per ml and incubated with 2.5 μM DiI-C16 (Molecular Probes, Invitrogen) for 30 min at 37°C, washed and 4x10^4^ cells per well were plated in the OrisTM Cell Migration Assay plates (Platypus Technologies) containing cell seeding stopper. Cells were allowed to attach and spread overnight at 37°C before the stoppers were removed and medium was refreshed. Migration was measured in real time using the POLARstar Omega plate reader (BMG LABTECH, Germany) with temperature at 37°C and 5% CO_2_ collecting data points every 20 min for up to 24 hours. Migration speed was measured by calculating the area under the curve for each well. Pictures were taken before and after the migration assays. Each treatment was done in triplicate.

### Continuous real-time cell analysis (RTCA) assay

The xCELLigence RTCA system (Roche Applied Science, Germany) is a quantitative assay that monitors changes in cell adhesion, spreading, morphology, and growth in real time. The system records electrical signals that denote cell impedance to the electrodes in each well and translates the signals to ‘cell index’ values, representing degree and intensity of cell adhesion, morphology, and growth. We used this system to examine cell adhesion and spreading of the MCF10A cells after knockdown of *MYO1C* as well as to examine a potential dose-dependent response to *MYO1C* depletion. The experimental setup included seeding 5,000 cells per well in 50 μl of growth media without antibiotic in microtiter E-plates (Roche Applied Science). Different mixtures of *MYO1C*-siRNA and scrambled siRNA were prepared in Lipofectamine RNAiMAX and Opti-MEM I reduced serum medium to yield final concentrations of 1, 3, 5, 10, 15 or 20 nM of *MYO1C*-siRNA and 50 μl of each mixture was added to wells containing 5,000 MCF10A cells. Western blot analysis of MYO1C protein levels at time point 48 post transfection showed concentrations 10, 15 and 20 nM of *MYO1C*-siRNA resulted in 90–95% MYO1C knock down, whereas concentrations 1, 3 and 5 nM of *MYO1C*-siRNA resulted in 75–80% MYO1C protein level reductions (data not shown). Accordingly, and to detect potential dose-dependent effects of *MYO1C* depletion on cell phenotypes as screened by the xCELLigence RTCA system, four different mixtures of *MYO1C*-siRNA and scrambled siRNA prepared in Lipofectamine RNAiMAX and Opti-MEM I reduced serum medium to yield final concentrations of 1, 3, 5, or 10 nM were used. Control wells received only 50 μl of the mock-transfecting mixture. The plates were placed onto the RTCA station in a humidified incubator at 37°C in which the cell index values per well were recorded every 15 min up to 72 hours. For each of the *MYO1C*-siRNA concentrations, mean values and standard error of the mean (SEM) were derived from six independent repeats.

### Serum activation assay

HeLa cells were seeded in 6-well plates and transfected the next day with either scrambled siRNA, serving as a control, or siRNA for *MYO1C* to a final concentration of 30 nM. After 48 hours’ incubation, the media were substituted with 2.5 ml per well new media without serum and antibiotics (PenStep), followed by incubation for another 24 hours. Subsequently, 500 μl serum was added into each well and cells were harvested and lyzed for protein extraction after 2, 5 and 20 min post serum activation. Three independent experiments were performed.

### Real-Time PCR (RT-PCR)

To verify the efficiency of knockdown of the siRNA against *MYO1C* in cell migration assays, RT-PCR was performed. RNA was isolated from cells using the RNeasy kit (74104, Qiagen, USA) and cDNA was made using the Taqman Reverse Transcription kit (4366596, Applied Biosystems by Life Technologies). RT-PCR was performed using probes from Universal ProbeLibrary (04683633001, Hoffmann-La Roche, Switzerland). Amplification and analysis was performed on an ABI 7900 (Applied Biosystems).

### SDS-PAGE and Western blotting

Cell lysates were prepared using supplemented RIPA buffer (89901, Thermo Scientific) and the protein concentrations were measured by Bradford protein assay (Bio-Rad Protein Assay Dye Reagent Concentrate 500–006, Bio-Rad Laboratories, USA) at 600 nm (Novaspec II, Biochrome, UK).

For Western blot, cell lysates containing 20 μg protein were denatured and resolved in pre-cast SDS-PAGE (NuPAGE 4–12% Bis-Tris gel, Invitrogen by Life Technologies). Gels were transferred using the iBlot system (IB3010-01; iBlot Transfer Stack, Regular-Nitrocellulose- and IB1001EU iBlot Gel Transfer Device, Invitrogen by Life Technologies). After fixation, membranes were blocked with 5% non-fat dry milk in TBST buffer at room temperature for 1 hour and immunoblotted by incubation with different optimized dilutions of monoclonal or polyclonal primary antibodies (Table 1). Secondary antibodies were horseradish peroxidase conjugated goat anti-rabbit IgG (H+L) (111-001-003) or goat anti-mouse IgG (H+L) (115-035-062, Jackson ImmunoResearch Laboratories, Inc., USA). Membranes were incubated in SuperSignal West Pico (34080) or Femto (34096) Chemiluminescent Substrate (1:1 mixture of Luminol/Enhancer and Stable Peroxide Buffer; Thermo Scientific Inc.) solutions 5 min prior to detection. Chemiluminescense signals were recorded with a LAS1000 camera (Luminescent Image Analyzer, LAS1000 Plus, Fuji-Film, Japan) using the Image Reader LAS1000 V2.6 program. Western blots were performed in independent triplicates for each experiment. Immunoblotting signals were quantified by ImageJ (http://rsbweb.nih.gov/ij) and protein levels were calculated as relative protein level normalized against Gapdh.

**Table 1:** List of primary antibodies and the optimized dilution.

Antibody	Dilution	Product number, Manufacturer
rabbit anti-AKT	1:500	• sc-8312, Santa Cruz Biotechnology • 9272, Cell Signaling Technology
rabbit anti-pAKT_S473_	1:500	sc-7985, Santa Cruz Biotechnology
rabbit anti-pAKT_T308_	1:500	• sc-16646, Santa Cruz Biotechnology • 4056, Cell Signaling Technology
rabbit anti-IRS-1	1:500	sc-7200, Santa Cruz Biotechnology
rabbit anti-MYO1C	1:600	HPA001768, Sigma-Aldrich
rabbit anti-ERK1/2	1:800	9102, Cell Signaling Technology
rabbit anti-phosphorylatedERK1/2	1:800	9101, Cell Signaling Technology
mouse anti-PTEN	1:500	sc-7974, Santa Cruz Biotechnology
mouse anti-RAS	1:200	80–1735, Assay Designs and Stressgen
rabbit anti-14-3-3β	1:500	sc-628, Santa Cruz Biotechnology
rabbit anti-GAPDH	1:500 to 1:1000	sc-25778, Santa Cruz Biotechnology
rabbit anti-p110α	1:500	• sc-7174, Santa Cruz Biotechnology • 4249, Cell Signaling Technology
rabbit anti-p110β	1:500	• sc-603, Santa Cruz Biotechnology • 3011, Cell Signaling Technology
rabbit anti-p110δ	1:500	sc-7176, Santa Cruz Biotechnology
mouse anti-p110δ	1:1000	p75220, BD Transduction Laboratories
rabbit anti-p85	1:500 1:1000	• sc-423, Santa Cruz Biotechnology • Q63787, Millipore

### Western blot analysis of PI3K/AKT and RAS/ERK pathways

One million HeLa cells were seeded in 60-mm petri dish format for 24 hours prior to the transfection. Cells were transfected with a total of 0.9 μg/ml of four different mixtures of the empty vector and *MYO1C*-construct, as described earlier, to produce final concentrations of *MYO1C*-construct (0.0, 0.3, 0.6 or 0.9 μg/ml) to investigate potential MYO1C dose-dependent effects in the present experimental setup. Cell lysates were prepared at 24 hours post transfection and subjected to Western blot analysis for MYO1C protein as well as 10 key components of the PI3K/AKT pathway (AKT, pAKT_S473_, pAKT_T308_, IRS-1, PTEN, 14-3-3β, p85, p110α, p110β, p110δ, Table 1) and three downstream proteins of the RAS/ERK signaling pathway (RAS, ERK and pERK). Information on the manufacturers from which the antibody orders were made as well as antibody dilutions used in the experiments are presented in Table 1.

### Statistical analysis

The statistical analysis software SPSS (Statistical Package for the Social Sciences; IBM SPSS Data Collection) version 21 was used. The data are presented as mean ± SEM (standard error of the mean). A probability value less than 0.05 were regarded as statistically significant.

## Results

### Protein levels of MYO1C was significantly lower in poorly differentiated endometrial carcinomas

**Fig 2:**
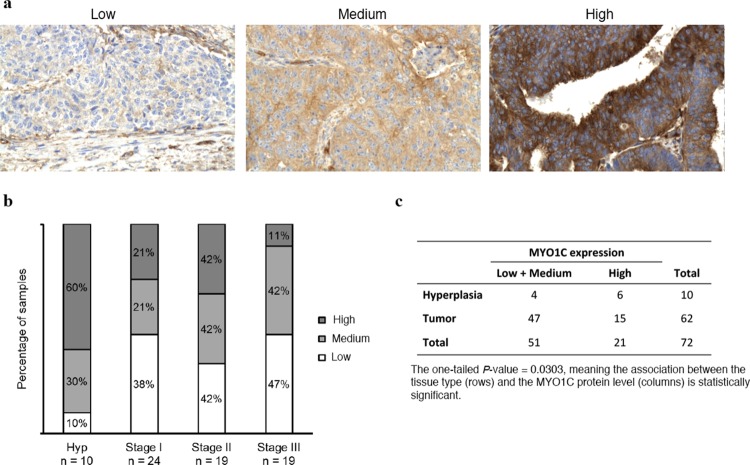
Protein levels of MYO1C in endometrial hyperplasia and endometrial adenocarcinomas of stage I-III. a) Representative images for the IHC staining of MYO1C in tissue samples of endometrial carcinoma with low, medium and high MYUO1C protein levels. b) Significant association was found between MYO1C protein level and tumor grade (*P* = 0.035, linear-by-linear association in the SPSS chi-square test). The proportion of samples with high MYO1C protein level was greatest in hyperplasias (Hyp) and smallest in stage III tumors, whereas the proportion of tumors with low abundance of MYOC1 was smallest in hyperplasias and highest in stage III tumors. Pairwise differences between sample groups were not significant. c) When the samples were regrouped as tumors and hyperplasia, the Fisher’s exact test showed significant difference in MYO1C expression between these two groups of samples (*P* = 0.0303).

Immunohistochemical analysis of a panel of 62 endometrial carcinomas (19 highly differentiated stage I tumors, 24 moderately differentiated stage II tumors, 19 poorly differentiated stage III tumors) and 10 endometrial hyperplasias revealed a significant association between MYO1C protein level and tumor grade (*P* = 0.035, linear-by-linear association in the SPSS chi-square test). In this analysis, we found that the proportion of samples with high level of MYO1C protein was highest in hyperplasias (60%) and lowest in stage III tumors (11%), whereas the proportion of tumors with low MYO1C protein level was lowest in hyperplasias (10%) and highest in stage III tumors (47%) (Fig 2B). Pairwise differences between sample groups were not significant; however, when the MYO1C protein level pattern in all tumors as a group (n = 62) were compared with hyperplasia samples (n = 10), the Fisher’s exact test analysis showed a statistically significant association (*P* = 0.0303) between groups of samples and levels of MYO1C (Fig 2C), indicating that the amount of MYO1C protein was significantly lower in endometrial carcinomas compared to hyperplasia.

### Over-expression of *MYO1C* resulted in significant decrease in cell proliferation

**Fig 3:**
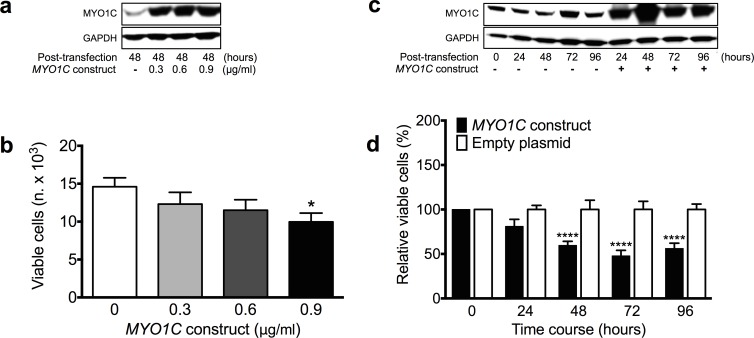
Over-expression of *MYO1C* significantly decreased viability of HEK-293 cells. (a) Immunoblot of MYO1C proteins in HEK-293 cells shows increased expression in the cells transfected with different amounts of *MYO1C*-construct at 48 hours post-transfection in comparison with the cells transfected with corresponding empty plasmid, serving as control. The image is a representative from at least three independent experiments. (b) Dose-response effect of over-expression of MYO1C protein on cell proliferation was observed at 48 hours post-transfection in comparison with cells transfected with empty vector. Each bar represents mean value ± *SEM* of all repeats, a Student’s *t*-test was used to compare the differences in mean values, *P-value < 0.05 versus the corresponding empty plasmid. (c) Immunoblot of MYO1C proteins in HEK-293 cells shows increased expression in *MYO1C*-transfected cells at 24, 48, 72 and 96 hours post-transfection. The image is a representative from at least three independent experiments. (d) Relative number of living cells was reduced in *MYO1C*-transfected cells. Significant decline was observed at 48, 72 and 96 hours post-transfection compared to cells transfected with the empty vector. Each bar represents mean value ± *SEM* of all repeats (at least three independent experiments, each in quadruplicates). A Student’s *t*-test was used to compare the differences in mean values, ****P-value < 0.001 versus the corresponding empty plasmid.

Using western blot, a panel of human cell lines, including embryonic non-cancerous HEK-293, adult non-cancerous MCF10A and adult cancerous HeLa cell lines were screened for *de novo* MYO1C protein expression (Fig 1). The choice for these cell lines was based on the feasibility of performing cell transfection experiments with these cells as well as to ensure different cell types (adult, embryonic, cancerous and normal cells) are tested. Cell lines with low (HEK-293 and HeLa) and medium to high (MCF10A) *de novo* MYO1C protein levels were selected for gene expression or knockdown experiments, respectively, to assess the effect of MYO1C on the cell proliferation. HEK-293 cells with low *de novo* MYO1C protein level were transfected with 0.0, 0.3, 0.6, or 0.9 μg/ml of the *MYO1C* gene expression construct (hereafter *MYO1C*-construct). The transfected cells were monitored for MYO1C protein level as well as for the number of live cells at 24, 48, 72 and 96 hours post transfection. Western blot showed a correlation between the amount of *MYO1C*-construct used in the cell transfection experiments and the levels of MYO1C protein in the cells (Fig 3A). Over-expression of MYO1C protein in the cells transfected with *MYO1C*-construct was observed at 24 hours and reached to its highest expression capacity after 48 hours (Fig 3B).

MTS-based cell proliferation assay was used to screen the number of live cells 48 hours after transfection with increasing amounts of *MYO1C*-construct (0.0, 0.3, 0.6 or 0.9 μg/ml), hence expressing increasing amount of MYO1C (Fig 3A). The analysis revealed a dose-dependent negative correlation between the amount of *MYO1C*-construct used in cell transfections and the number of live cells (Fig 3B). This dose-dependent and negative correlation reached a significant level (*P* < 0.05) between the control cells (received 0.0 μg/ml *MYO1C*-construct) and those transfected with the highest concentration (0.9 μg/ml) of the *MYO1C*-construct (Fig 3B).

Based on results from this screening experiment, and to ensure cells express enough amount of MYO1C protein to produce significant effects, we chose the concentration 0.9 μg/ml *MYO1C*-construct for the cell proliferation assays. Cells were transfected with 0.9 μg/ml of *MYO1C*-construct or the empty vector and were monitored for the number of live cells and protein level of MYO1C at 24, 48, 72 and 96 hours post transfections. Western blot analysis revealed successful expression of MYO1C protein following gene construct transfection in the cells at 24 hours, reaching its highest capacity after 48 hours (Fig 3C). The analysis revealed a strong response in the cells to MYO1C over-expression in form of a highly significant (*P* < 0.001) decrease in the number of live cells starting at 48 hours post transfection and throughout the experiment time span (Fig 3D).

### *MYO1C*-siRNA treatment resulted in significant increased cell proliferation

**Fig 4:**
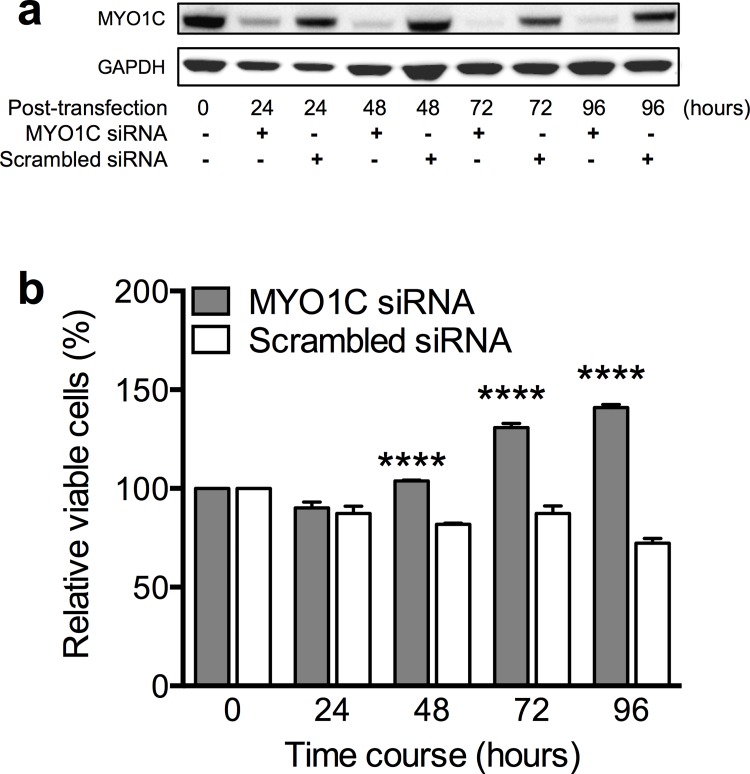
siRNA-knockdown of *MYO1C* enhanced viability. (a) Immunoblot of MYO1C protein expression in MCF10A cells transfected with *MYO1C*-siRNA and negative control (scrambled siRNA) to the final concentration of 10nM at 24, 48, 72 and 96 hours post-transfection. The image is a representative from at least three independent experiments. (b) Histogram showing percentages of relative cell viability of MCF10A cells transfected with *MYO1C*-siRNA and scrambled siRNA to the final concentration of 10nM. Significant increase in the number of live cells was observed at 48, 72 and 96 hours post-transfection compared to cells transfected with the scrambled siRNA. Each bar represents mean value ± *SEM* of all repeats (at least three independent experiments, each in quadruplicates). A Student’s *t*-test was used to compare the differences in mean value, ****P-value < 0.001 versus cells treated with scrambled siRNA as well as untreated cells (time point zero).

We next aimed to confirm the observed effect of *MYO1C* expression on reduced number of live cells in a reciprocal experimental design using a *MYO1C* mRNA silencing approach. For this experiment we used normal breast epithelial MCF10A cells with high *de novo* MYO1C protein levels, as identified by the initial Western blot screening of human cell lines (Fig 1). MCF10A cells were transfected with 10 nM *MYO1C*-specific siRNA or scrambled siRNA followed by MTS-based cell proliferation screening at 24, 48, 72 and 96 hours post transfections. Western blot analysis revealed successful reduction in MYO1C protein levels in the *MYO1C*-siRNA transfected cells with the maximal effect (over 90% suppression) at 72 and 96 hours post-transfection compared to cells that received scrambled siRNA (Fig 4A). *MYO1C*-siRNA cell proliferation assays indicated again a strong negative correlation between the level of MYO1C protein expressed and the number of live cells. This negative correlation became highly significant (*P* < 0.001) at 48, 72 and 96 hour time points post transfection compared to untreated cells (time point zero, Fig 4B).

### Reduced *MYO1C* expression impaired cell migration, cell spreading and cell adhesion

To investigate the potential effect of *MYO1C* on cell migration, cell spreading and cell adhesion, we used *MYO1C*-siRNA treatment in two independent experimental setups: the gap closure assay and xCELLigence RTCA system.

**Fig 5:**
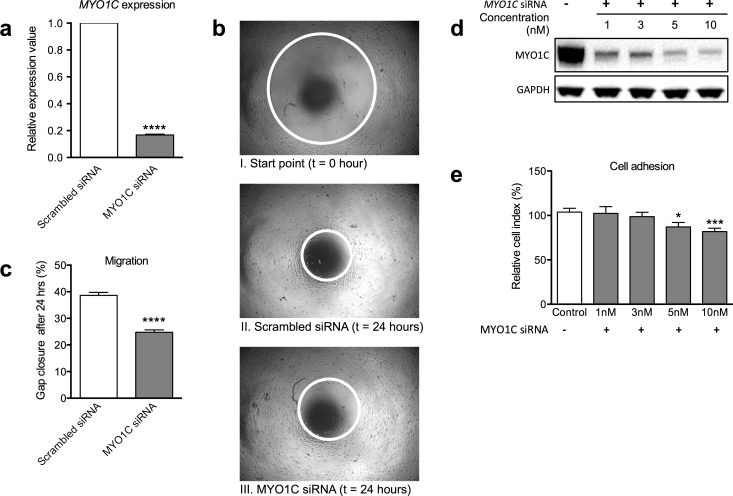
Decrease in cell migration and adhesion of MCF10A cells after knockdown of *MYO1C*. (a) The expression levels of *MYO1C* in the MCF10A cells subjected to 20 nM siRNA transfection were verified with RT-PCR. (b) Geometry on gap immediately after ripping of the stopper (I) and geometry on gap of the control (scrambled siRNA) (II), and *MYO1C*-siRNA transfected (III) after 24-hour incubation were shown. The white circles show the area of the geometry on gap at each measurement. (c) The analyses of the geometry on gap closure (migration assay) of MCF10A cells transfected with *MYO1C*-specific siRNA was evaluated after ripping off cell seeding stopper followed by 24-hour incubation in comparison to the geometry of control cells. (d) Immunoblot of MYO1C protein levels in MCF10A cells transiently transfected with different concentrations of *MYO1C*-siRNA (1, 3, 5 and 10 nM) and control (mock transfection) at 48 hours post-transfection. (e) Histogram showing percentages of relative cell index of MCF10A cells transfected with *MYO1C*-siRNA and control cells (received mock transfection). A dose-dependent decrease in relative cell index response to MYO1C depletion was observed reaching to a significant value with concentrations of 5 and 10 nM of *MYO1C*-siRNA at 48 hours post-transfection compared to control cells (mock transfection). Images shown in (b) and (d) are representatives from three and six independent experiments, respectively. Each bar in (a), (c) and (e) represents the mean value ± *SEM* from three and six independent experiments, respectively. A Student’s *t*-test was used to compare the differences in mean value, *P-value < 0.05, ***P-value < 0.005 and ****P-value < 0.001 versus control cells.

MCF10A cells with high *de novo* protein expression of MYO1C were transfected with 20 nM of *MYO1C*-siRNA or scrambled siRNA (efficiency of siRNA treatment was first controlled by RT-PCR, Fig 5A) and the cells were subjected to the gap closure assay. In this assay, cells are allowed to attach to the plate containing a cell seeding stopper and spread overnight, a gap in the well is created by removing the cell seeding stopper, cells are allowed for another 24 hours to move into the gap to fill the empty space and a plate reader measures the geometry of the gap in real time (Fig 5B). The analyses revealed that MCF10A cells transfected with *MYO1C*-siRNA, hence with significant diminished expression of *MYO1C*, were significantly slower in moving and closing the produced gap compared to the control cells (Fig 5C).

In another set of experiments, we used the xCELLigence RTCA system to monitor for the potential alteration in the adhesion and spreading capacity of MCF10A cells in response to different concentrations of *MYO1C*-siRNA treatment. This experiment was performed to address three research questions: to verify results from the gap closure assay, to investigate whether the observed impaired cell mobility in response to *MYO1C* depletion in gap closure assay is due to impaired cell adhesion and spreading, and to examine whether the response to *MYO1C* depletion follows a dose-dependent pattern. The RTCA assay is a highly sensitive and quantitative system that provides a continuous (real-time) cell monitoring system in which the electrode impedance to the cell mobility and adherence is measured and displayed as a cell index. The impedance is dependent on the number of cells as well as the intensity of interaction of cells with the electrodes, reflected by the ability of cells to proliferate and to adhere to the surface of electrodes, respectively.

After an initial screening experiment, MCF10A cells were transfected with 1, 3, 5, or 10 nM of *MYO1C*-siRNA and cells were subjected to analysis with the RTCA system every 15 min for up to 72 hours. The efficiency of the treatment was verified by Western blot at 48 hours post transfection showing a linear correlation between *MYO1C*-siRNA concentration and MYO1C protein depletion (from 75% to over 90% depletion, Fig 5D). The analysis revealed lowered expression of MYO1C resulted in diminished cell indexes and this reduction was suggested to be dose-dependent as it reached a significant level (*P* < 0.05) for the concentration 5.0 nM treatment and a highly significant (*P* < 0.001) for the concentration 10.0 nM treatment compared to the control cells treated with the mock transfection (Fig 5E).

### Over-expression of MYO1C protein resulted in higher p110α protein levels, and decreased PTEN, AKT and pAKT levels

**Fig 6:**
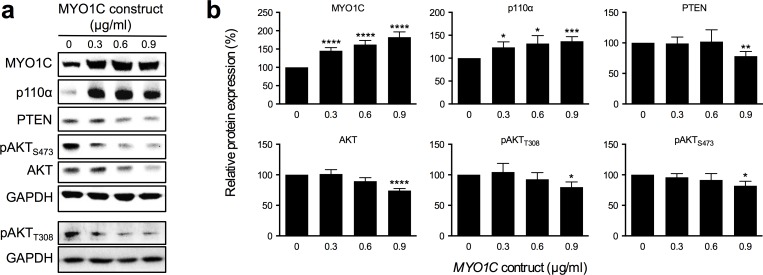
Increased expression of MYO1C protein affects expression of PI3K/AKT components. (a) The immunoblot of proteins from cell lysates of the HeLa cells transfected with serial dilution of *MYO1C*-gene expression constructs and (b) derived relative protein expressions in response to serial transfection of *MYO1C* that showed a decreasing in AKT, pAKT_S473_, pAKT_T308_ and PTEN at the maximal dose but increasing in p110α protein expression in comparison with cells transfected with empty construct (control); each bar represents mean value (± SEM), a Student’s *t*-test was used to compare the differences in mean value, *P-value < 0.05, ***P-value < 0.005 and ****P-value < 0.001 versus control cells. The images shown are representative of Western blotting from at least three independent experiments.

To investigate the potential involvement of *MYO1C* in the PI3K/AKT and RAS/ERK signaling pathways, we examined potential alterations of eight key components of the PI3K/AKT signaling pathway (AKT, pAKT_S473_, pAKT_T308_, IRS-1, PTEN, 14-3-3β, p85, p110α/β/δ) and three downstream components of the RAS/ERK signaling pathway (RAS, ERK and pERK) in response to induced over-expression of MYO1C protein in cells transfected with the *MYO1C*-construct. Cells were transfected with four different concentrations (0.0, 0.3, 0.6 or 0.9 μg/ml) of *MYO1C*-construct, cell lysates were prepared at 24 hours post transfections and subjected to Western blot analysis to investigate potential dose-dependent effect of MYO1C over-expression on expression, stabilization and/or activation of these proteins. The analysis revealed a dose-dependent response to MYO1C over-expression in the cells that reached its maximum in response to 0.9 μg/ml *MYO1C*-construct transfection as follows (Fig 6A and 6B):

- Significant decrease in the expression levels of the total AKT protein (*P* < 0.0001) and both of its phosphorylated forms (pAKT_S473_, pAKT_T308_) (*P* < 0.05 and *P* < 0.05).
- Significant decrease in the expression level of the PTEN protein (*P* < 0.001).
- Significant increase (*P* < 0.0001) in the p110α protein level.
- No significant change in the expression level/activation of the remaining seven proteins was detected.

### Decreased *MYO1C* expression allowed fast serum-induced activation of AKT

**Fig 7:**
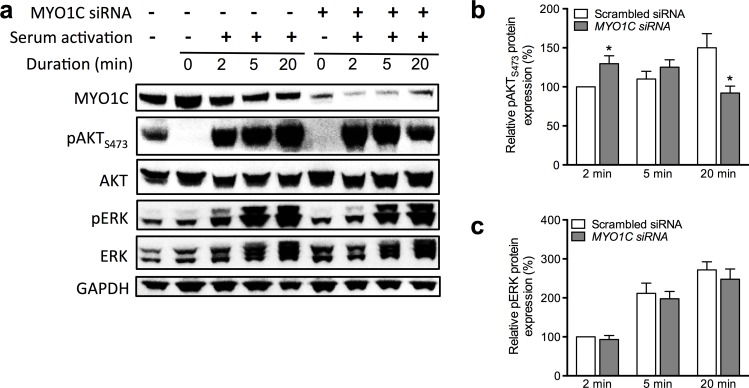
Fast activation of AKT in response to serum activation after knockdown of *MYO1C* in HeLa cells. (a) The immunoblot of proteins from cell lysates of the HeLa cells transfected with *MYO1C*-siRNA in comparison with non-transfected HeLa cells and subjected to serum activation for 2, 5 and 20 minutes. Histogram showing percentages of relative protein expression of pAKT_S473_ (b) and pERK (c) from HeLa cells after serum supplement for 2, 5 and 20 minutes. The expression of pAKT_S473_ was significantly higher after serum supplement at 2 minutes and significantly lower after 20 minutes in comparison to control cells (scrambled siRNA); there was no significant difference in activation of ERK. Each bar in (b) and (b) represents the mean value ± *SEM* for at least three independent repeats. A Student’s *t*-test was used to compare the differences in mean value, *P-value < 0.05 versus control cells. The images shown are representative of Western blotting and serum activation assay from at least three independent experiments.

We next investigated potential effect of depletion of MYO1C protein (Fig 7A) on serum-induced activation (phosphorylation) of AKT and ERK in the cells treated with increasing amount of *MYO1C*-siRNA. This experiment was designed with a double goal: 1- to confirm and verify the observed negative correlation between the MYO1C protein level and phosphorylation of AKT (Fig 6) and 2- to clarify whether the observed lowered level of pAKT_S473_ in response to over-expression of MYO1C (as presented in Fig 6) was a natural consequence of decreased expression level of AKT or was rather a specific response to the MYO1C over-expression.

Cells transfected with different concentrations of *MYO1C*-siRNA or scrambled siRNA were serum starved for 24 hours, serum was added to the cell cultures and at 2 min, 5 min and 20 min after addition of serum, levels of pAKT and pERK in the cell lysates were examined. The analysis showed a very low level of pAKT_S473_ in both cells transfected with *MYO1C*-siRNA or scrambled siRNA (control) after 24-hour serum starvation. Following serum stimulation, in cells transfected with *MYO1C*-siRNA it was found that AKT was rapidly (within 2 min) phosphorylated in its residue S473 (pAKT_S473_), whereas in the control cells transfected with scrambled siRNA this effect was significantly less (Fig 7B). Interestingly, the pattern of serum-induced AKT activation appeared to be completely different between the *MYO1C*-siRNA transfected cells and the scrambled siRNA-transfected control cells: in the control cells the level of pAKT_S473_ slowly, but stably increased, whereas in the *MYO1C*-siRNA treated cells there was a fast (at 2 min) and significantly strong AKT activation response to the serum stimulation that over the time went in the other direction and reached a significant reduced level at 20 min post serum stimulation (Fig 7B). No significant correlation between the expression level of MYO1C and phosphorylation of ERK, before and after serum treatment, was detected (Fig 7C).

## Discussion

In the year 2000, Hanahan and Weinberg proposed six hallmarks of cancer (revised later to eight hallmarks); eight traits that are required for a cell to evolve from normal to tumor: ability to sustain proliferative signaling, ability to evade growth suppressors, ability to avoid apoptosis, ability to induce angiogenesis, ability to replicate immortally, ability to avoid immune destruction, ability to deregulate cellular energetics, and ability to invade and metastasize [37, 38]. In the present work, by using clinical samples along with cell culture-based functional assays, we aimed to examine the original hypothesis of *MYO1C* acting as a tumor suppressor gene [6] in relation to some of Hanahan and Weinberg’s proposed tumorigenesis abilities.

We examined levels of MYO1C protein in a panel of well-stratified endometrial carcinomas and found a significant negative association (*P* = 0.035) between the level of MYO1C protein expression in hyperplasia and stage III tumors (Fig 2). Pairwise analysis of differences between stage I, II and III sample groups did not reach significant values, indicating that lowered protein level of MYO1C is equally important in all tumor stages. We also found significant (*P* = 0.0303) association comparing sample type (tumor vs hyperplasia) and MYO1C protein level, indicating that the level of MYO1C protein is significantly lower in tumors compared to hyperplasia samples. In conclusion, our analysis revealed a significant association between lowered MYO1C protein level and endometrial carcinogenesis and also indicated that reduced MYO1C levels is equally important in early as well as late tumor stages, suggesting an important role for MYO1C in all stages of endometrial carcinogenesis.

We then performed cell transfection experiments with *MYO1C*-over expression constructs or *MYO1C*-siRNA to over-express or knockdown MYO1C protein in the cells, respectively, followed by cell proliferation assays. Our analysis revealed that cells expressing extra MYO1C protein had a diminished cell proliferation capacity (Fig 3). This finding was amply validated in a reverse experiment design in which *MYO1C* gene expression was knocked down using siRNA, where we detected a significant correlation between reduced MYO1C protein level and increased number of viable cells (Fig 4). Taken together, these experiments suggest a significant role for the MYO1C protein in cell proliferation, presumably through regulation of one of or a combination of the first three of Hanahan and Weinberg’s proposed tumorous traits: ability to sustain proliferative signaling, ability to evade growth suppressors, and/or ability to avoid apoptosis [37, 38].

The tumorous trait of “ability to evade growth suppressors” can be reached through impaired cell adhesion/cell spreading-mediated contact inhibition [39] and/or becoming irresponsive to cell proliferation regulation mechanisms [40, 41]. Accordingly, we next aimed to examine the potential role of MYO1C protein in cell adhesion, spreading and migration. We performed two independent cell migration/cell adhesion/cell spreading experiments, the OrisTM Cell Migration Assay (gap closure assay) and the xCELLigence Real-Time Cell Analyzer (RTCA) system, for cells in which *MYO1C* expression was effectively knocked down using the siRNA technology (Fig 5A). These parallel experiments jointly confirmed that reduction in MYO1C protein resulted in a significant reduced ability of the cells to adhere to the surface, spread and therefore to migrate (Fig 5B–5D). Cells characteristically can spread on an extracellular matrix by extending membrane protrusions such as membrane ruffles, and can adhere to other cells and their surrounding stroma via specific contact sites known as focal adhesions [42]. The contact with the surrounding cells, matrix and surfaces is one of the limiting factors that determine how and to what extent a normal cell can grow and divide. In fact loss of “contact inhibition” is recognized as one of the strategies adapted by the tumorous cells towards acquiring the “ability to evade growth suppressors” [37, 38]. Moreover, repetition of cycles of cell spreading and adhesion processes on opposing sides of a cell forms the basis for cellular migration [43], which in turn relies on lipid rafts [44]. The lipid raft’s role in this context is to concentrate the signaling molecules necessary for migration/spreading on the cell surface, thereby efficiently and rapidly promoting the corresponding signal transductions [45]. It has been shown that MYO1C specifically and tightly binds to PI(4,5)P2 (PIP2) [17, 18], is spatially associated with lipid rafts [19], is involved in regulation of lipid raft recycling, and therefore may contribute to signaling pathways underlying cell migration and spreading. Using the Cell Tracker CMRA system combined with velocity tracking software analysis, Brandstaetter et al. (2012) showed that knockdown of MYO1C dramatically reduces cell spreading and causes a reduction in lipid raft markers at the cell surface of spreading HeLa cells. MYO1C knockdown also resulted in the redistribution of focal adhesions, as well as in a reduction in the migration speed and track length of migrating cells [19, 46]. Here we report a similar observation, but using different methodologies and a different cell line (MCF10A). Taken together, in the present work we provided additional evidence for potential contribution of tumor suppressor candidate MYO1C to mechanism/s involved in cell adhesion, spreading and migration, confirming and extending the earlier findings. Here we hypothesize that reduced expression of tumor protein MYO1C results in impaired cell adhesion and cell spreading-mediated contact inhibition, and therefore in induced tumorous phenotype.

Several reports indicate a role for MYO1C in the glucose uptake into adipocyte and muscle cells through insulin-induced tethering of glucose transporter 4 (GLUT4) vesicles [12, 23, 47, 48] potentially through the PI3K/AKT signaling pathway [49]. Beside glucose homeostasis, the PI3K/AKT signaling is known as one of the main signaling pathways involved in cell survival, proliferation and apoptosis, and thus contributes to cancer development. In the present work we aimed to investigate the potential interplay between MYO1C and expression and/or activation of components of the PI3K/AKT signaling pathway.

When cells were transfected with a gene expression *MYO1C-*construct, in Western blot analysis of the transfected cells for 11 components from PI3K/AKT and RAS/ERK signaling pathways, we detected significant increase in the expression level of p110α protein, significant decrease in the expression level of the PTEN protein and in the expression levels of the total AKT protein as well as both of its phosphorylated forms (Fig 6A and 6B). Our analysis provided evidence for a potential interplay between MYO1C and PI3K/AKT signaling in cells that are not stimulated.

Catalytic subunits of PI3K play a critical role in growth factor signaling and survival by phosphorylating inositol lipids. Gain of function mutations in the *PIK3CA* gene (encoding p110α) has been found to be oncogenic and is implicated in cervical cancers [50]. It is shown that p110α is involved in the activation of AKT upon stimulation by receptor tyrosine kinases ligands such as EGF, insulin, IGF-1, VEGFA and PDGF [51, 52]. In the present work we found a significant positive correlation between levels of MYO1C and p110α in cells that were not stimulated. Accordingly, the observed level of p110α in the cells is not reflecting the activation status of PI3K/AKT signaling, but could reflect a potential involvement of MYO1C in *PIK3CA* gene expression. An alternative explanation for the observed effect could be a protective feedback mechanism in response to MYO1C-induced PI3K/AKT suppression, in response to which cells produce excess of p110α to be able upon growth factor stimulation to rapidly counteract MYO1C function.

Among PtdIns(3,4,5)P_3_ phosphatases, *PTEN* (phosphatase and tensin homologue deleted on chromosome 10) has long been identified as a haploinsufficient tumor suppressor gene and negative regulator of the insulin signaling via PI3K/AKT signaling by dephosphorylating PIP_3_ [53, 54]. Expression of PTEN inhibits PI3K signaling through rapid decrease of elevated insulin-induced PtdIns(3,4,5)P_3_ levels, thus inhibiting the phosphorylation of downstream targets such as AKT [55]. Here we found that in response to excess expression of MYO1C, the level of PTEN protein was reduced in the cell. However, as cells were at resting (not stimulated) state, we hypothesize that the observed effect could be a gene expression feedback system, i.e. in the presence of MYO1C, there is less need for the PI3K/AKT modulating PTEN protein, hence *PTEN* gene expression has become down-regulated.

In the MYO1C over-expression experiments, we additionally detected a strong and significant negative correlation between protein levels of MYO1C and AKT and also a weaker negative correlation, but still significant, between levels of MYO1C and pAKT_S473_ and pAKT_T308_ (Fig 6B). As the cells were not stimulated in this experiment, the negative correlation between MYO1C expression and levels of pAKT_S473_ and pAKT_T308_ reflects a potential inhibitory role for MYO1C protein on expression as well as activation of AKT. As in this experiment a very strong negative correlation between MYO1C and AKT protein levels was detected, this raised the question whether the detected reduced levels of pAKT_S473_ and pAKT_T308_ were a direct outcome of reduced expression of AKT protein, rather than MYO1C inhibitory function on AKT activation. To address this, we performed serum starvation/stimulation experiments to examine the dynamic of AKT activation in response to *MYO1C*-siRNA treatment. In this experiment we observed a significantly stronger and rapid pAKT_S473_ response to serum stimulation in cells lacking MYO1C compared to the control cells (Fig 7B). This fast response was time limited and the level of pAKT_S473_ rapidly diminished, reaching a significantly lower level compared to the control cells after 20 min (Fig 7B). In this experiment we chose to examine only the pAKT_S473_ response, as the basal phosphorylation at residue S473 is the initial step for activation of AKT and only after this basal activation can the AKT protein become fully activated through the second phosphorylation at its residue T308.

MYO1C is shown to tightly bind to Rictor, which is a downstream protein in the PI3K/AKT pathway that can make a complex with the mammalian target of rapamycin complex 2 (mTORC2). This complex is responsible for the basal activation of AKT through phosphorylation of its Serine 473 residue [56, 57]. Interestingly, in their study, Hagen et al. concluded that the Rictor-mTORC2 complex is independent of the Rictor-MYO1C complex, and also that MYO1C was not required for mTORC2-dependent AKT activation in 3T3-L1 adipocytes [56, 57]. They found that with increasing doses of insulin, Rictor knockdown cells showed a reduction in the level of pAKT_S473_ compared to control cells, whereas MYO1C-depleted cells did not show a similar effect [56, 57]. It is of note that Hagan et al. looked at the insulin stimulation effects on transfected cells at only one time point (15 min). In our study, using a different cell type (MCF10A), we could clearly show that depletion of MYO1C resulted in a rapid increased level of pAKT_S473_ at 5 min post stimulation that was significantly reduced after 20 min. Accordingly, it is possible that the previous studies [56, 57] with their experimental design missed the strong and rapid activation of AKT in response to MYO1C depletion.

Here we hypothesize that in the presence of MYO1C, as it tightly binds to Rictor, the Rictor-mTORC2 complex may not fully function, hence phosphorylation of AKT at its Serine 473 residue is become impaired (Fig 6). With the same logic, in the absence of MYO1C, the Rictor protein is set free to make a complex with mTORC2 and thus to phosphorylate AKT (Fig 7A and 7B). However, our experiment design suggests that the dynamic between Rictor-mTORC2 and Rictor-MYO1C complexes may not be as simple as proposed above. In cells expressing normal levels of MYO1C (Fig 7B, white bars), over time a linear increase of the pAKT_S473_ levels in response to the serum stimulation was observed. However, in cells lacking MYO1C (Fig 7B, dark bars), there was a fast pAKT_S473_ response that rapidly went to the other direction and after 20 min reached a significantly lower level in cells with diminished MYO1C compared to control cells. It appears in the MYO1C depleted cells the hyper-activated Rictor-mTORC2 complex is becoming quickly exhausted and not able to exert its function as efficiently over time. Whether the presence of MYO1C is required for the stability of Rictor and/or stability of the Rictor-mTORC2 complex is an interesting research question for further investigation. Taken together, our results suggest that normal levels of MYO1C are required for normal function of the Rictor-mTORC2 complex and when MYO1C is lacking or its expression is disturbed, the AKT signaling is deregulated in favor of hyper-activation of PI3K/AKT signaling, and towards tumorous features.

## Conclusion

Results from the present work provide evidence for inhibitory function of *MYO1C* on cell proliferation, presumably through repression of PI3K/AKT signaling, hence supporting the initial hypothesis of tumor suppressor activity for this gene. We additionally showed that reduced *MYO1C* expression resulted in diminished cell migration and impaired cell adhesion; phenotypes that may contribute to loss of “contact inhibition”. These outcomes are additional supporting evidences for the proposed tumor suppressor characteristic of *MYO1C*. Finally, we found that the MYO1C protein level was reduced in endometrial carcinomas and that this reduced expression was significantly different between tumors and hyperplasia samples.

Over-activation of the PI3K/AKT signaling pathway contributes to the development of most cancer types, hence identification of newly described tumor suppressor gene associated with this pathway would offer novel routes of cancer treatment strategies. Further studies are required to fully understand details of the contribution of *MYO1C* to carcinogenesis and to address the above-mentioned hypotheses and questions.
